# Multiple Sclerosis in Older Adults: The Clinical Profile and Impact of Interferon Beta Treatment

**DOI:** 10.1155/2015/451912

**Published:** 2015-04-01

**Authors:** Afsaneh Shirani, Yinshan Zhao, John Petkau, Paul Gustafson, Mohammad Ehsanul Karim, Charity Evans, Elaine Kingwell, Mia L. van der Kop, Joel Oger, Helen Tremlett

**Affiliations:** ^1^Department of Medicine, Division of Neurology and Brain Research Centre, UBC Hospital, University of British Columbia, 2211 Wesbrook Mall, Vancouver, BC, Canada V6T 2B5; ^2^Department of Statistics, University of British Columbia, 3182 Earth Sciences Building, 2207 Main Mall, Vancouver, BC, Canada V6T 1Z4; ^3^College of Pharmacy and Nutrition, University of Saskatchewan, 110 Science Place, Saskatoon, SK, Canada S7N 5C9; ^4^Department of Public Health Sciences, Karolinska Institutet, 171 77 Stockholm, Sweden

## Abstract

*Background*. We examined (1) patient characteristics and disease-modifying drug (DMD) exposure in late-onset (LOMS, ≥50 years at symptom onset) versus adult-onset (AOMS, 18–<50 years) MS and (2) the association between interferon-beta (IFN*β*) and disability progression in older relapsing-onset MS adults (≥50 years). *Methods*. This retrospective study (1980–2004, British Columbia, Canada) included 358 LOMS and 5627 AOMS patients. IFN*β*-treated relapsing-onset MS patients aged ≥50 (regardless of onset age, 90) were compared with 171 contemporary and 106 historical controls. Times to EDSS 6 from onset and from IFN*β* eligibility were examined using survival analyses. *Results*. LOMS patients (6%) were more likely to be male, with motor onset and a primary-progressive course, and exhibit faster progression and were less likely to take DMDs. Nonetheless, 57% were relapsing-onset, of which 31% were prescribed DMDs, most commonly IFN*β*. Among older relapsing-onset MS adults, no significant association between IFN*β* exposure and disability progression was found when either the contemporary (hazard ratio [HR]: 0.46; 95% CI: 0.18–1.22) or historical controls (HR: 0.54; 95% CI: 0.20–1.42) were considered. *Conclusion*. LOMS differed clinically from AOMS. One-third of older relapsing-onset MS patients were prescribed a DMD. IFN*β* exposure was not significantly associated with reduced disability in older MS patients.

## 1. Introduction

Multiple sclerosis (MS) is a chronic degenerative disease of the central nervous system. It is said to be the most common cause of neurological disability in young adults in the Western world [1]; however, as life expectancy is only minimally affected [2], the impact of disability can be felt across a relatively long lifespan. Clinical symptoms characteristically first present in people in their mid-20s to 30s; however, a wide spectrum exists, and as the general population ages, the prevalence of MS in older adults rises [3]. Despite the average age of those living with MS in western countries having reached an all-time high (mid-50s) [3], relatively little is known about the clinical characteristics of “older” adults with MS or their use of and response to treatment.

While some previous studies have described the characteristics and/or prognosis of older MS patients [4–12], we were unable to find any studies examining exposure rates or response to disease-modifying drugs (DMDs) in these patients. Historically, older adults were actively excluded from internationally recognized diagnostic criteria for MS (e.g., Poser criteria) [13], as well as from clinical trials. For example, the most widely used DMDs for relapsing-onset MS were approved based on the pivotal clinical trials where the average age of patients was 35 and those over the age of 50 or 55 were excluded [14–16]. Therefore, there remains a need to better understand this understudied, older population with MS.

Here, we first describe the demographic and clinical characteristics, as well as the prescription patterns, of DMDs in patients with late-onset MS (LOMS) defined as symptoms onset after 50 years of age in comparison with adult-onset MS (AOMS) patients; and second we examine the association between exposure to interferon beta (IFN*β*) and disability progression in relapsing-onset MS patients aged ≥50 (regardless of onset age) in the real-world setting in British Columbia (BC), Canada.

## 2. Methods

### 2.1. Design and Setting

We conducted a retrospective cohort study based on prospectively collected data in the British Columbia Multiple Sclerosis (BCMS) database. Established in 1980, the BCMS database is estimated to capture 80% of the British Columbian MS population [17, 18] and links the four MS clinics in BC over the study period (1980–2004). The database has been used extensively for research [10, 19–21]. The study was approved by the University of British Columbia's Clinical Research Ethics Board, which includes informed patient consent.

### 2.2. Patients and Data

Patients had to be registered with a BCMS clinic between August 1980 and December 2004 and diagnosed with definite MS (based on Poser or McDonald criteria) [13, 22]. For aim 1, that is, description of those with LOMS versus AOMS, LOMS was defined as symptom onset at age of 50 or older and AOMS as symptom onset between 18 and <50 years of age. Clinical and demographic data, including onset symptoms, relapses in the first 5 years, disability (Expanded Disability Status Scale [EDSS]) at first clinic visit, and DMD exposure, were derived from the BCMS database, with follow-up until December 31, 2008. Onset symptoms were classified as motor, sensory, and optic neuropathy and cerebellar, ataxic, or brainstem (CAB) and initial disease course as relapsing versus primary progressive [23].

For aim 2, that is, investigation of the association between exposure to IFN*β* and disability progression, older adults aged 50 or older at baseline, that is, at eligibility for IFN*β* treatment (regardless of onset age), were potentially considered. This broader age criterion was specific to aim 2 to make the findings more clinically relevant to daily practice and to maximize the cohort size. IFN*β* drugs were selected since they represent the most commonly used DMDs. IFN*β* eligibility was broadly adapted [19] from the BC government's reimbursement scheme which required patients to have definite relapsing-onset MS and an EDSS score ≤6.5. An EDSS score of 6.5 indicates the need for constant bilateral assistance to walk about 20 meters without resting [24]. Study baseline was defined as the first clinic visit following their 50th birthday at which a patient reached eligibility for IFN*β* treatment between April 1985 and December 2004. April 1985 was the first date that health-related administrative data became available. These data were accessed to provide individual patient-level data on IFN*β* prescription (through the province's comprehensive PharmaNet database) [25]; information on preexisting comorbidities (through hospital and community (physician visit) data, that is, the Hospital Separations Discharge Abstract [26] and Medical Service Plan Payment [27] data); and socioeconomic status (through Census Geodata and geocodes provided by the Medical Services Plan Registration & Premium Billing file) [28]. Clinical data were linked with these health administrative data using each patient's unique personal health number, facilitated by Population Data BC (a panprovincial population health data resource). Patients were excluded if they had fewer than two prospective EDSS assessments from baseline or were enrolled in a clinical trial or exposed to DMDs prior to study baseline. From the population of eligible patients, three cohorts were selected: one treated cohort and two separate untreated control cohorts (one contemporary and one historical). The treated cohort included patients exposed to IFN*β* who first became treatment eligible between July 1995 (when the first IFN*β* was licensed for MS in Canada) and December 2004. The contemporary control cohort comprised patients who first became treatment eligible in the same period (July 1995–December 2004) but who remained unexposed to IFN*β*. The historical control cohort included those first eligible prior to the approval of IFN*β* (April 1985–June 1995) but who remained unexposed to IFN*β* throughout the study period. Patients were followed until the last EDSS assessment prior to the study end date (December 31, 2008). Patients eligible for the current study would also have been included in a wider study examining the association of IFN*β* exposure and disability progression in MS [19], but that study was not specifically designed to study IFN*β* exposure in older adults with MS.

### 2.3. Exposure to IFN*β* Treatment

To examine the association between exposure to IFN*β* treatment and disability progression (aim 2), all IFN*β* products were grouped together as one therapeutic class, including IFN*β*-1b [Betaseron, 250 *μ*g subcutaneously on alternate days] and IFN*β*-1a [Avonex, 30 *μ*g intramuscularly once weekly; and Rebif, 22 *μ*g or 44 *μ*g subcutaneously 3 times per week] [29]. Since 98% of exposed patients had either no break or a break of <3 months between consecutive administrations of IFN*β* over the study period, product switches or breaks were not considered as treatment interruptions for these analyses.

### 2.4. Outcomes

The main disability outcome was time to a confirmed and sustained EDSS score of 6, from MS symptom onset (for aim 1) and from IFN*β* eligibility date for aim 2. EDSS 6 indicates “intermittent or unilateral constant assistance (cane, crutch, and brace) required to walk about 100 meters with or without resting” [24]. EDSS 6 was defined as “confirmed” when a subsequent score of ≥6 at least 150 days later was recorded and “sustained” when all subsequent EDSS scores were ≥6. The secondary outcome for aim 2 was time from baseline to a confirmed and sustained score of 4. An EDSS score of 4 indicates being “fully ambulatory without aid, up and about 12 hours a day despite relatively severe disability; able to walk without aid 500 meters” [24].

### 2.5. Statistical Analyses

For aim 1, demographic and clinical characteristics and DMD prescription patterns for the LOMS versus AOMS cohorts were compared using the Pearson *χ* ^2^ test for categorical variables and the *t*-test for continuous variables. The Kaplan-Meier survival curves and the log-rank test were used to compare time from MS onset to confirmed and sustained EDSS 6. The independent effect of potential risk factors on time to reach EDSS 6 was examined using a multivariable Cox regression model, with sex, onset symptoms, and onset age category (AOMS versus LOMS), with relapsing-onset and primary-progressive MS patients examined separately. Two sensitivity analyses were conducted. Firstly, patients with an unknown time to EDSS 6 (because EDSS 6 had already been reached at the first clinic visit) were included by imputing the midway time between MS onset and the first clinic assessment. Secondly, data were censored once a DMD was initiated.

For aim 2, similar bivariate statistics were used when comparing the baseline characteristics of the IFN*β*-treated versus contemporary and historical untreated cohorts, along with the Mann-Whitney-Wilcoxon test for ordinal variables. The Kaplan-Meier survival curves were used to estimate the proportion of patients reaching the main outcome within 10 years of study baseline. Multivariable Cox proportional hazards regression models were used to assess the hazard of reaching EDSS 6 and 4, with IFN*β* exposure as a time-dependent predictor (to minimize immortal time bias) [30]. By considering exposure to IFN*β* as a binary time-dependent variable, we were able to adjust for changes in treatment status. More specifically, the value of this binary predictor changed according to the patient's treatment status at each event time and thus accounted for the unexposed time from baseline to initiation of IFN*β*, the actual IFN*β*-exposed time, and the unexposed time between stopping IFN*β* and the end of follow-up. The main model was also adjusted for sex, age, disease duration, and EDSS score at baseline in all analyses. Additional model adjustments included annualized relapse rate (based on the two years prior to baseline) and, for the contemporary approach only, socioeconomic status and preexisting comorbidities (because of data availability). The proportional hazard assumption was assessed using log-log plots. Results were expressed as hazard ratios with 95% confidence intervals (CI).

All statistical tests were 2-sided, and *P* < 0.05 was considered statistically significant. Statistical analyses were conducted using the Statistical Package for the Social Sciences (SPSS Inc. Chicago, Illinois, version 16.0).

## 3. Results

For aim 1, 5985 patients were included, 358 (6%) with LOMS and 5627 (94%) with AOMS. Patient characteristics (clinical and demographic) are shown in Table 1. Those with LOMS were more likely to be male, present with motor or CAB symptoms, and have a primary-progressive course at onset, whereas they were less likely to present with sensory or optic neuropathy symptoms compared to AOMS patients. Exposure to MS drugs was less common in LOMS patients (19.6% versus 38.1% for AOMS), although a reasonable proportion of relapsing-onset LOMS patients were exposed (30.6% versus 41.0% of those with AOMS).

**Figure 1:**
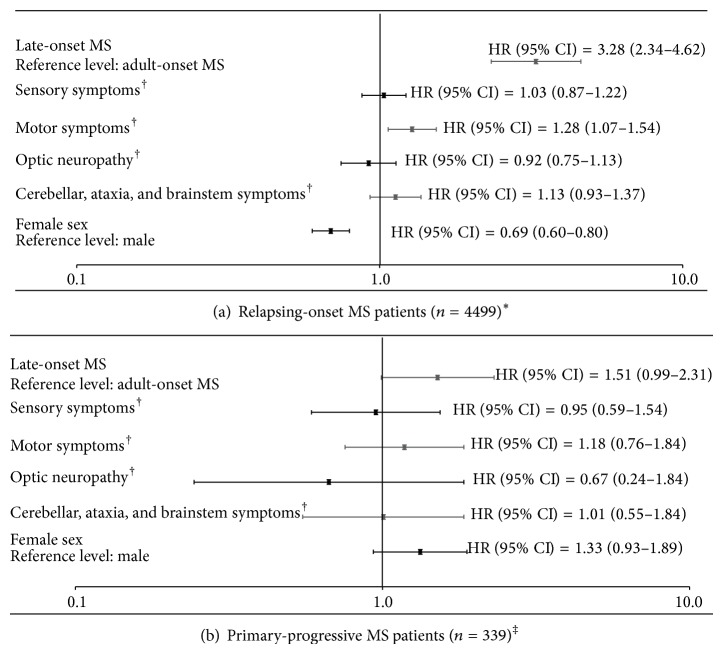
Multivariable Cox regression analysis of potential factors associated with time to reach confirmed and sustained EDSS 6 from onset of MS symptoms in patients with relapsing-onset (a) and primary-progressive (b) MS.  ^∗^Out of 5373 patients with relapsing-onset MS, 874 patients did not contribute to the analysis (i.e., were excluded from the analyses). Those included 640 patients who had already reached the outcome by first clinic assessment (i.e., were left-censored), 185 patients with no EDSS score recorded, and 49 patients who were censored before the earliest event. ^†^Reference level = absence of the specific onset symptom. ^‡^Out of 612 patients with primary-progressive MS, 273 patients did not contribute to the analysis (including 234 patients who had already reached the outcome by first clinic assessment (i.e., left-censored), 33 patients with no EDSS score recorded, and 6 patients who were censored before the earliest event).

Patients with either relapsing-onset or primary-progressive LOMS (versus AOMS) progressed more rapidly from onset to EDSS 6 (Supplementary Figure e-1 in Supplementary Material available online at http://dx.doi.org/10.1155/2015/451912). After adjusting for sex and onset symptoms, the hazard of progression to EDSS 6 in the relapsing-onset cohort was 3.28 times higher (95% CI: 2.34–4.62) for LOMS versus AOMS patients and 1.51 times higher (95% CI: 0.99–2.31) in the primary-progressive cohort (although the 95% CI included one); see Figure 1. Findings from the sensitivity analyses were in a similar direction to those observed in the main analyses (Figures e-2 to e-5).

**Figure 2:**
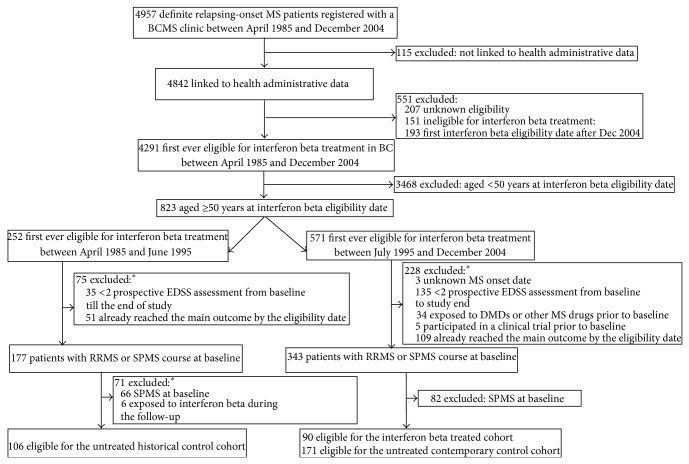
Selection of the interferon beta-treated and untreated cohorts aged ≥50 at interferon beta eligibility date. BCMS clinic, British Columbia Multiple Sclerosis clinic; DMD, disease-modifying drug; EDSS, Expanded Disability Status Scale; RRMS, relapsing-remitting multiple sclerosis; and SPMS, secondary progressive multiple sclerosis.  ^∗^The sum of the individual reasons (numbers) exceeds the total number of patients in some boxes because some patients met more than one condition.

For aim 2, a total of 367 patients were included (Figure 2). From these, 90 formed the IFN*β*-treated cohort, 171 formed the contemporary control cohort, and 106 formed the historical control cohort. Their baseline characteristics are shown in Table 2. The proportion of women in each cohort ranged from 70.2% to 81.1%, with more women in the treated versus contemporary untreated cohort. The mean age at MS onset was similar between the three cohorts, ranging from 40.7 to 43.3 years. The mean baseline disease duration was shorter in the treated cohort (10.9 ± 10.7 years) compared to the untreated contemporary (14.9 ± 11.4 years) and historical (15.1 ± 12.1 years) cohorts; however the median baseline EDSS score was 2.5 in all the three cohorts.

**Figure 3:**
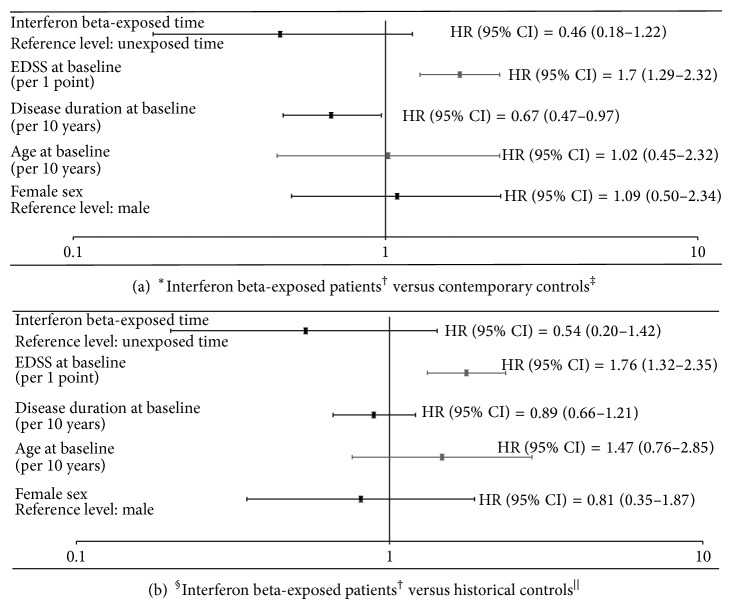
Multivariable time-dependent Cox regression analysis of potential factors affecting time to reach confirmed and sustained EDSS 6 for 90 interferon beta-exposed patients versus 171 contemporary controls aged ≥50 at baseline, with interferon beta treatment as a time-varying covariate (a). Results of the same analysis for 90 interferon beta-exposed patients and 106 historical controls (b).  ^∗^Ten patients who were censored before the earliest event did not contribute to the analysis. ^†^266 person-years of interferon beta exposure and 151 person-years of untreated time (92 person-years before and 59 person-years after the initiation of interferon beta treatment). ^‡^597 person-years of untreated time. ^§^Seven patients who were censored before the earliest event did not contribute to the analysis. ^||^694 person-years of untreated time.

Findings from the adjusted multivariable Cox regression model, with IFN*β* exposure as a time-dependent covariate, are shown in Figure 3. We found no significant association between exposure to IFN*β* treatment and time till progression to the main outcome, sustained and confirmed EDSS 6, when either of the contemporary or historical control cohorts was the comparison group (HR = 0.46, 95% CI: 0.18–1.22 for the contemporary approach [Figure 3(a)] and HR = 0.54, 95% CI: 0.20–1.42 for the historical approach [Figure 3(b)]). In both approaches, a higher baseline EDSS was associated with a higher hazard of reaching EDSS 6. Adding the annualized relapse rate (based on the two years prior to baseline) to the model did not change findings in either the contemporary or historical approach (HR = 0.48, 95% CI: 0.18–1.26 and HR = 0.51, 95% CI: 0.19–1.33, resp.), nor did adding comorbidity or socioeconomic status (data not shown). The actual numbers of individuals that reached the main outcome were 10 (11.1%), 22 (12.9), and 31 (29.2%) in the treated, contemporary control, and historical control cohorts, respectively. The estimated proportions of patients (from Kaplan-Meier curves) reaching the main outcome within 10 years of baseline were 31.1%, 30.3%, and 39.5%, respectively. Findings were also similar when the secondary outcome (EDSS 4) was considered (see Figure e-6).

## 4. Discussion

In our cohort, over 1 in 16 adults with MS had symptom onset at or after 50 years of age. These patients differed clinically from those with AOMS. Consistent with other studies [6, 10–12], there were proportionally more men with motor onset symptoms and a primary-progressive course in the LOMS (versus AOMS) cohort. However, unlike some other studies [6, 10], we still observed that the majority of LOMS cohort had a relapsing-onset course of which nearly one-third were exposed to a disease-modifying drug for MS. Among older adults with relapsing-onset MS, we found no significant association between exposure to the most commonly used DMD, IFN*β*, and progression of disability. To our knowledge, this is the first time a substantial cohort of relapsing-onset patients with LOMS have been described clinically in the posttreatment era.

Overall, 6% of our adult cohort fulfilled the criteria for LOMS; others using a similar definition have reported 5%–12% affected [11, 31]. Why there are such differences between studies is not clear, although possible reasons could include study design, cohort size, case ascertainment, life expectancy in the underlying population, and genetic susceptibility. There can be challenges to determining disease course in older adults, including a higher chance of an extended subclinical period or possibly overlooked relapsing-remitting disease activity prior to the overt clinical manifestation of MS [32], such that it is possible that the proportion of LOMS patients with a relapsing-onset course is underestimated. Nonetheless, our observations are of importance given that the only DMDs licensed to date for MS are for patients with a relapsing-onset course.

Our study greatly expands on a previous study examining the natural history of LOMS [10]. By nearly tripling our LOMS cohort size, we were able to more precisely determine the difference in disease progression for relapsing-onset MS patients, with the hazard of reaching EDSS 6 being threefold higher for those with LOMS versus AOMS [10].

We were able to find few other studies describing DMD exposure in LOMS with which to compare our findings [6]. One German study identified 52 patients with LOMS (defined as aged >50 at MS diagnosis) and reported a lower rate of exposure to DMDs in LOMS patients compared to those with a younger-onset MS (under 40 years at diagnosis) [6]. Similar to our findings, the most frequently used DMD was IFN*β*, with 10% of those with LOMS exposed versus 54% in the younger-onset cohort.

The treatment of older adults with MS can be challenging. There is an absence of evidenced-based guidelines; older people in general are often underrepresented in pharmaceutical clinical trials. The pivotal clinical trials of IFN*β* drugs excluded those over the age of 50 or 55 [14–16]. While later clinical trials have increased the upper age limit to 60 or 65 years old [33–35], only one of these focused on IFN*β*, with eligibility restricted to those with secondary-progressive MS [35]. Further barriers to including older patients in clinical trials can relate to preexisting comorbidities which may be more prevalent in these patients. This paucity of evidence highlights the importance of observational studies such as ours.

Information on the impact of DMDs on older MS patients is crucial for a number of reasons. Firstly, older adults with MS are typically treated using therapeutic guidelines originally established for younger adults; however, there is yet no direct evidence to support this practice [32]. Secondly, as aging profoundly affects the pharmacodynamics and pharmacokinetics of drugs as well as the immune system, it is not unreasonable to speculate that immunomodulatory therapies may have a different effect in older MS patients. In addition, the average follow-up time in clinical trials is typically too short (2-3 years) [14–16] to capture the longer-term progression profile of patients. The clinical trial setting is also different from that of the real-world condition in other aspects such as patients' comorbidities and motivation or ability to adhere to medications.

Strengths of our study included a sizable cohort of LOMS patients, inclusion of those with AOMS as a comparison group, substantial follow-up time, use of a conservative definition of the progression outcome, linkage to health-related administrative datasets to provide a rich data source, the selection of a comparable baseline (eligibility for IFN*β* treatment) for the treated and control cohorts, consideration of treatment exposure as a time-varying covariate thereby addressing immortal time bias [30] and accounting for the changing treatment status of patients over the follow-up period, and inclusion of both pre- and post-IFN*β* era control cohorts thereby addressing indication bias.

Our study has also some limitations. An estimated 20% of the MS patients in BC are not captured in the database. Since we included only patients who were attending a BCMS clinic, it is possible that those with LOMS could be over- or underrepresented. EDSS, despite being the most widely used scale for measuring MS disability, is weighted toward physical disability and overlooks other relevant aspects of MS such as cognitive dysfunction. LOMS is associated with more rapid progression, increasing the chances of reaching EDSS 6 before first visit in the clinic (“left-censoring”); however we were able to show through a sensitivity analysis that this did not change our main findings. For aim 2, we were not able to examine possible differences between the various IFN*β* products. We were not able to consider biomarkers, imaging data, ethnicity, and the possible effect of change in diagnostic criteria from Poser to McDonald in our study. Our study was not designed to look into the safety profile of IFN*β* drugs which may differ between LOMS and AOMS patients. Our study was also not designed to examine whether the disease course has changed over time (drug treatments aside). This was addressed in a previous study, in which we observed MS disease progression to remain relatively stable in BC over two decades [36]. As with any observational study, unmeasured confounding remains possible. Finally, the confidence intervals around our hazard estimates were such that we cannot rule out definitively that the IFN*β*s could delay progression in older adults; there is a real need for further studies in this area.

To the best of our knowledge, this is the largest study of its kind to examine LOMS and the first designed specifically to examine the association between exposure to IFN*β* and disability progression in older MS patients in the real-world setting. Given that the prevalence of older people living with MS will continue to increase as the underlying populations age, there is a real need to better understand the characteristics of these patients and potential response to drug treatments for MS. Ultimately, as many of these patients will be treated with DMDs in clinical practice, there is an urgent need for further studies to develop evidence-based therapeutic guidelines for this special population.

## Supplementary Material

In this supplementary material, we present the results of additional analyses including comparison of disability progression from onset to EDSS 6 in patients with either relapsing-onset or primary progressive late-onset MS versus adult-onset MS, as well as several sensitivity analyses to account for patients with an unknown time to EDSS 6 (because EDSS 6 had already been reached at the first clinic visit), and to account for the effect of censoring data once a disease modifying drug was initiated.

## Figures and Tables

**Table 1:** Characteristics of patients with late-onset versus adult-onset MS, British Columbia, Canada [aim 1].

Characteristics	Late-onset MS (*n* = 358)	Adult-onset MS (*n* = 5627)	*P* value
Sex, *n* (%)			
Male	132 (36.9)	1550 (27.5)	<0.001^∣∣^
Female	226 (63.1)	4077 (72.5)	
Age at onset, mean (±SD), median [range], years	55.4 (4.9), 54.1 [50.0–75.8]	32.0 (8.0), 31.2 [18.0–49.99]	<0.001^¶^
*n* (%)	50–<55 yrs: 201 (56.1)	18–<30 yrs: 2539 (45.1)
	55–<60 yrs: 104 (29.1)	30–<40 yrs: 2013 (35.8)
	≥60 yrs: 53 (14.8)	40–<50 yrs: 1074 (19.1)
Onset symptoms, *n* (%)			
Motor			
Present	140 (39.1)	1042 (18.5)	<0.001^∣∣^
Absent	218 (60.9)	4585 (81.5)	
Sensory			
Present	107 (29.9)	2564 (45.6)	<0.001^∣∣^
Absent	251 (70.1)	3063 (54.4)	
Optic neuropathy			
Present	20 (5.6)	949 (16.9)	<0.001^∣∣^
Absent	338 (94.4)	4678 (83.1)	
Cerebellar, ataxia, or brainstem			
Present	71 (19.8)	849 (15.1)	0.016^∣∣^
Absent	287 (80.2)	4778 (84.9)	
Initial course, *n* (%)			
Relapsing	206 (57.5)	5167 (91.8)	<0.001^∣∣^
Primary-progressive	152 (42.5)	460 (8.2)	
Age at first visit^∗^, mean (±SD), median [range], years	60.5 (6.4), 59.5 [50.4–81.0]	41.1 (10.3), 40.7 [18.4–80.9]	<0.001^¶^
Disease duration at first visit^∗^, mean (±SD), median [range], years	5.2 (4.7), 3.7 [0.03–30.0]	9.1 (8.9), 6.2 [0.0–59.2]	<0.001^¶^
Disease duration at last visit^∗^, mean (±SD), median [range], years	10.2 (5.7), 9.7 [0.3–30.3]	16.9 (10.4), 15.1 [0.00–60.2]	<0.001^¶^
Length of prospective follow-up time^∗†^, mean (±SD), median [range], years	5.0 (4.2), 4.1 [0–19.3]	7.8 (6.5), 6.4 [0–28.4]	<0.001^¶^
Annualized relapse rate during the first five years after onset of symptoms^‡^, mean (±SD), median [range]	0.2 (0.3), 0.2 [0–1.8]	0.3 (0.4), 0.2 [0–3.2]	0.109^¶^
Year of registration with the clinic, *n* (%)			
1980–1985	51 (14.2)	979 (17.4)	0.355^∣∣^
1986–1990	46 (12.8)	799 (14.2)	
1991–1995	63 (17.6)	992 (17.6)	
1996–2000	103 (28.8)	1568 (27.9)	
2001–2004	95 (26.5)	1289 (22.9)	
Exposure to a “disease modifying drug” for MS, *n* (%)			
Ever	70 (19.6)	2143 (38.1)	<0.001^∣∣^
Never	288 (80.4)	3484 (61.9)	
Exposure to a “disease modifying drug” for MS among patients with a relapsing-onset course only, *n* (%)			
Ever	63 (30.6)	2120 (41.0)	0.003^∣∣^
Never	143 (69.4)	3047 (59.0)	
Initially prescribed drug among patients with a relapsing-onset course only, *n* (%)			
Interferon beta-1a (intramuscular)	4 (6.3)	274 (12.9)	
Interferon beta-1a (subcutaneous)	22 (34.9)	731 (34.5)	
Interferon beta-1b (subcutaneous)	24 (38.1)	755 (35.6)	
Glatiramer acetate	11 (17.5)	254 (12.0)	
Natalizumab	0 (0)	9 (0.004)	
Mitoxantrone	1 (1.6)	43 (2.0)	
Others^§^	1 (1.6)	54 (2.5)	

EDSS, Expanded Disability Status Scale.

^∗^Missing for 56 patients with adult-onset MS.

^†^Time from first clinic visit to most recent clinic visit.

^‡^Only reported for patients with relapsing-onset MS with at least 5 years of follow-up after the onset of symptoms including 4578 patients with adult-onset MS and 163 with late-onset MS.

^§^Others included clinical trial drugs and cytotoxic immunosuppressants: teriflunomide (both cohorts) and in the adult-onset cohort, methotrexate, azathioprine, cyclophosphamide, FTY720 (fingolimod), cladribine, Hu23F2G (rovelizumab), lenercept, MBP8298 (dirucotide), NBI-5788, paclitaxel, and interferon alpha.

^∣∣^Pearson's chi-square test.

^¶^Student's *t*-test.

**Table 2:** Characteristics of the treated and untreated (contemporary and historical) cohorts [aim 2]. All comprise relapsing-onset MS patients aged ≥50 at interferon beta eligibility date (baseline).

Characteristics (at baseline^∗^, unless otherwise stated)	Interferon beta-treated patients (*n* = 90)	Contemporary untreated patients^†^ (*n* = 171)	*P* value^‡^	Historical untreated patients^§^ (*n* = 106)	*P* value^∣∣^
Sex, *n* (%)					
Male	17 (18.9)	51 (29.8)	0.06^¶^	24 (22.6)	0.52^¶^
Female	73 (81.1)	120 (70.2)		82 (77.4)	
Age at MS onset, years (mean ± SD)	43.3 ± 11.6	40.7 ± 11.1	0.08^**^	41.0 ± 11.7	0.16^∗∗^
*n* (%)					
<30	13 (14.4)	36 (21.1)	0.31^¶^	20 (18.9)	0.36^¶^
30–<40	19 (21.1)	38 (22.2)		22 (20.8)	
40–<50	27 (30.0)	55 (32.2)		39 (36.8)	
≥50	31 (34.4)	42 (24.6)		25 (23.6)	
Disease duration, years (mean ± SD; median [range])	10.9 ± 10.7; 7.5 [0.03–43.5]	14.9 ± 11.4; 13.3 [0.2–53.3]	0.007^∗∗^	15.1 ± 12.1; 12.4 [0.3–47.9]	0.01^∗∗^
Age, years (mean ± SD)	54.2 ± 4.5	55.6 ± 4.9	0.03^∗∗^	56.1 ± 5.5	0.01^∗∗^
*n* (%)					
50–<55	65 (72.2)	95 (55.6)	0.03^¶^	55 (51.9)	0.01^¶^
55–<60	16 (17.8)	54 (31.6)		28 (26.4)	
≥60	9 (10.0)	22 (12.9)		23 (21.7)	
EDSS score (mean ± SD; median [range])	2.5 ± 1.2; 2.5 [0–6]	2.5 ± 1.2; 2.5 [0–6.5]	0.99^††^	2.4 ± 1.2; 2.5 [0–6]	0.62^††^
Annualized relapse rate in the two years prior to baseline^‡‡^ (mean ± SD)	0.6 ± 0.7	0.4 ± 0.6	0.006^∗∗^	0.4 ± 0.6	0.08^∗∗^
Active follow-up time (first to last EDSS assessment), years (mean ± SD)	5.1 ± 2.7	4.3 ± 2.7	0.03^∗∗^	8.6 ± 5.1	<0.001^∗∗^
Charlson comorbidity index^§§^ (median [range])					
*n* (%)	0 [0-1]	0 [0–3]		—^∣∣∣∣^	
0 (no comorbidity)	85 (94.4)	164 (95.9)	0.59^¶^		
≥1 (at least one comorbid condition)	5 (5.6)	7 (4.1)			
Neighbourhood income quintile^¶¶^, *n*(%)					
1 (lowest income)	12 (13.6)	33 (20.2)	0.21^¶^	—^∣∣∣∣^	
2	11 (12.5)	33 (20.2)			
3	16 (18.2)	28 (17.2)			
4	19 (21.6)	30 (18.4)			
5 (highest income)	30 (34.1)	39 (23.9)			

EDSS, Expanded Disability Status Scale.

^∗^Baseline was considered as the first date a patient became eligible for interferon beta treatment (whilst being >=50 years old).

^†^Untreated patients who first became eligible for treatment in the “interferon beta era” (whilst being >=50 years old).

^‡^Comparison of the interferon beta-treated patients with the contemporary untreated patients.

^§^Untreated patients who first became eligible for treatment in the “pre-interferon beta era” (whilst being >=50 years old).

^∣∣^Comparison of the interferon beta-treated patients with the historical untreated patients.

^¶^Pearson's chi-square test.

^∗∗^Student's *t*-test.

^††^Mann-Whitney-Wilcoxon test.

^‡‡^If this period included MS onset, the first onset attack was not included as a relapse.

^§§^Deyo adaptation of the Charlson comorbidity index, based on hospital admissions or physician visits in the two years prior to baseline and derived from International Classification of Diseases, 9th Revision, Clinical Modification (ICD-9-CM) codes, excluding hemiplegia, paraplegia, and dementia to avoid misclassifying complications of MS as comorbidity. All relevant comorbidities were aggregated into a single variable theoretically ranging from 0 to 33; higher scores indicate more comorbidity.

^∣∣∣∣^Data incomplete/unavailable.

^¶¶^Used as a proxy for socioeconomic status. Data were missing for 2 patients in the interferon beta-treated cohort and 8 patients in the contemporary control cohort.
