# Supplementary material for: Multiple Sclerosis in Older Adults: The Clinical Profile and Impact of Interferon Beta Treatment
Source: Biomed Res Int. 2015 Apr 1;2015:451912. doi: 10.1155/2015/451912 (PMC4397470; doi:10.1155/2015/451912)
Supplement: Supplementary file 1 — In this supplementary material, we present the results of additional analyses including comparison of disability progression from onset to EDSS 6 in patients with either relapsing-onset or primary progressive late-onset MS versus adult-onset MS, as well as several sensitivity analyses to account for patients with an unknown time to EDSS 6 (because EDSS 6 had already been reached at the first clinic visit), and to account for the effect of censoring data once a disease modifying drug was initiated. [file 451912.f1.pdf]

Supplementary web-appendix

Supplement to: Shirani A, Zhao Y, Petkau J, Gustafson P, Karim ME, Evans C, Kingwell E, van der Kop ML, Oger J, Tremlett H. Multiple sclerosis in older adults: the clinical profile and impact of interferon beta treatment.

.

Contents

Supplementary Figure e-1
Supplementary Figure e-2
Supplementary Figure e-3
Supplementary Figure e-4
Supplementary Figure e-5
Supplementary Figure e-6

Panel A: Relapsing-onset MS patients (n=4548)*

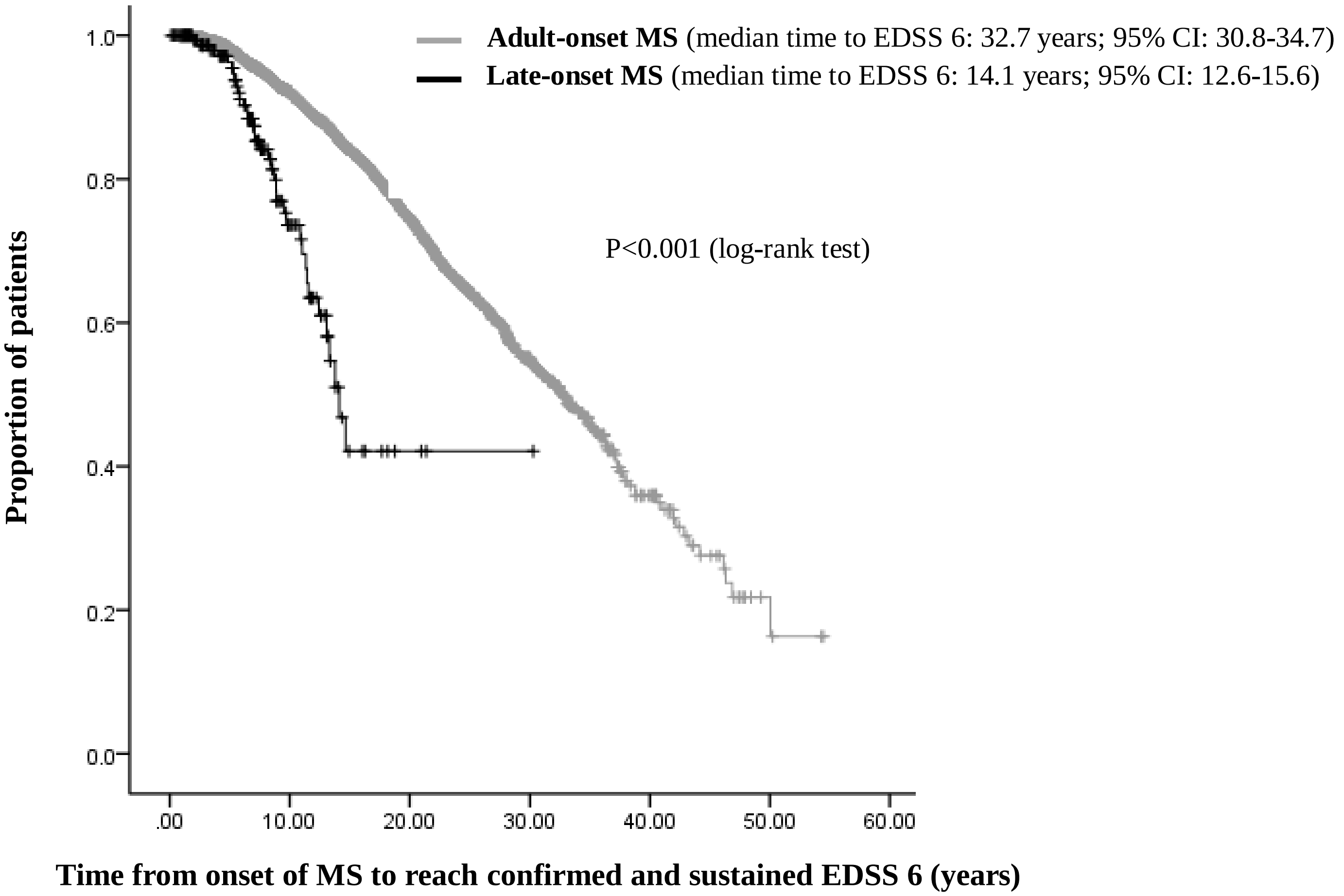

Patients at risk of progressing to EDSS 6 at selected time points

MS onset	Time from onset of MS (years)					Right-censored patients†
	0	5	10	15	20	
**Adult-onset**	4381	3672	2579	1662	1004	3523 (80.5%)
**Late-onset**	167	116	41	8	3	131 (78.4%)

Figure e1. Panel A

Panel B: Primary-progressive MS patients (n=345)[‡]

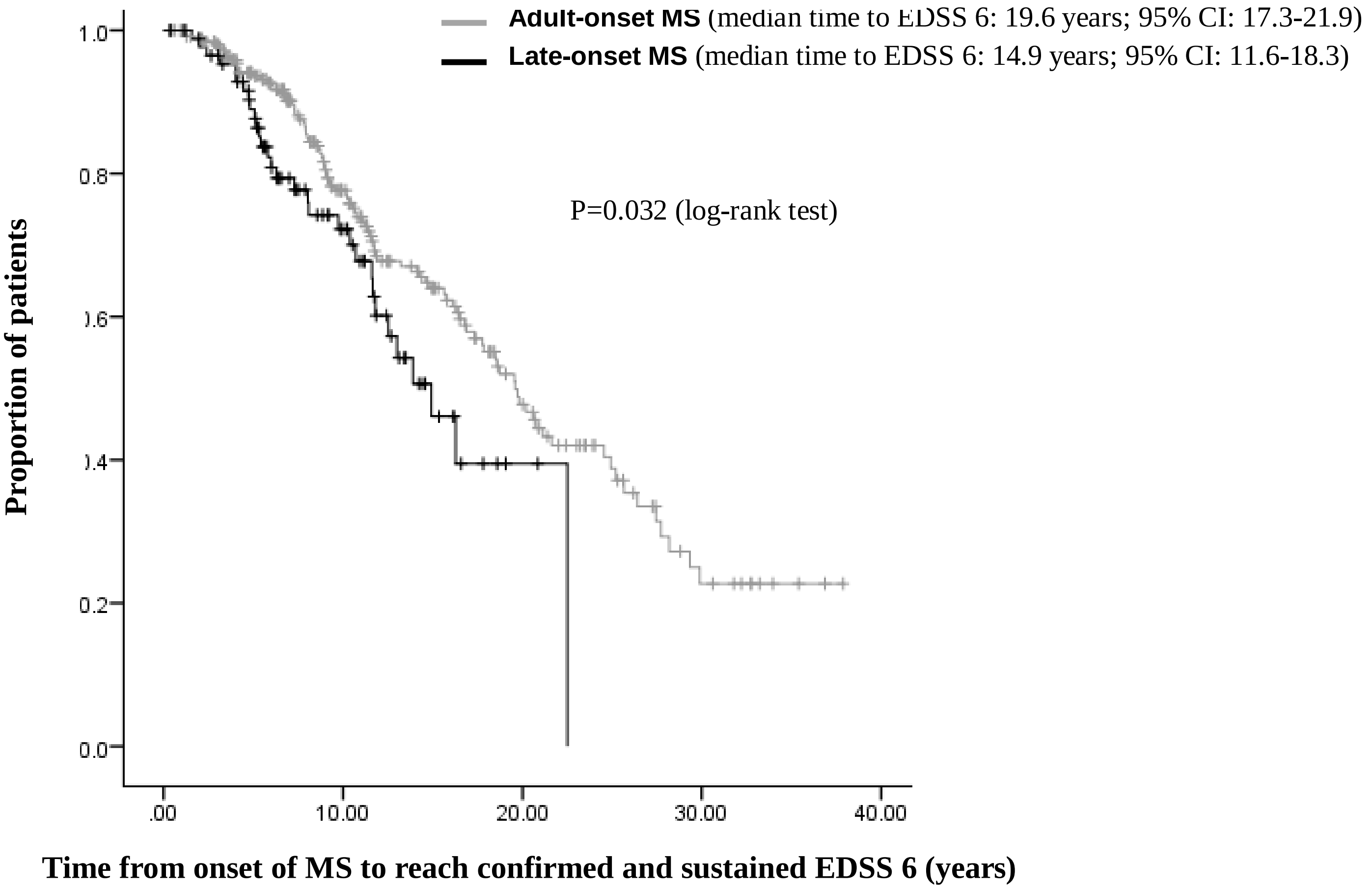

Patients at risk of progressing to EDSS 6 at selected time points

MS onset	Time from onset of MS (years)					Right-censored patients[†]
	0	5	10	15	20	
**Adult-onset**	255	203	128	80	45	258 (62.0%)
**Late-onset**	90	69	35	10	2	59 (65.6%)

Figure e1. Panel B

Figure e1. Kaplan-Meier survival curves showing time to reach confirmed and sustained EDSS 6 from onset of MS symptoms for adult-onset vs. late-onset MS patients, by disease course (Panel A: relapsing-onset MS patients; Panel B: primary-progressive MS patients).

[*] Out of 5373 patients with relapsing-onset MS, 825 patients who had already reached the outcome by first clinic assessment did not contribute to the analysis (i.e. were left-censored).

[†] Those who did not reach EDSS 6 during the follow-up period.

[‡] Out of 612 patients with primary-progressive MS, 267 patients who had already reached the outcome by first clinic assessment did not contribute to the analysis (i.e. were left-censored).

Panel A: Relapsing-onset MS patients (n=5188)*

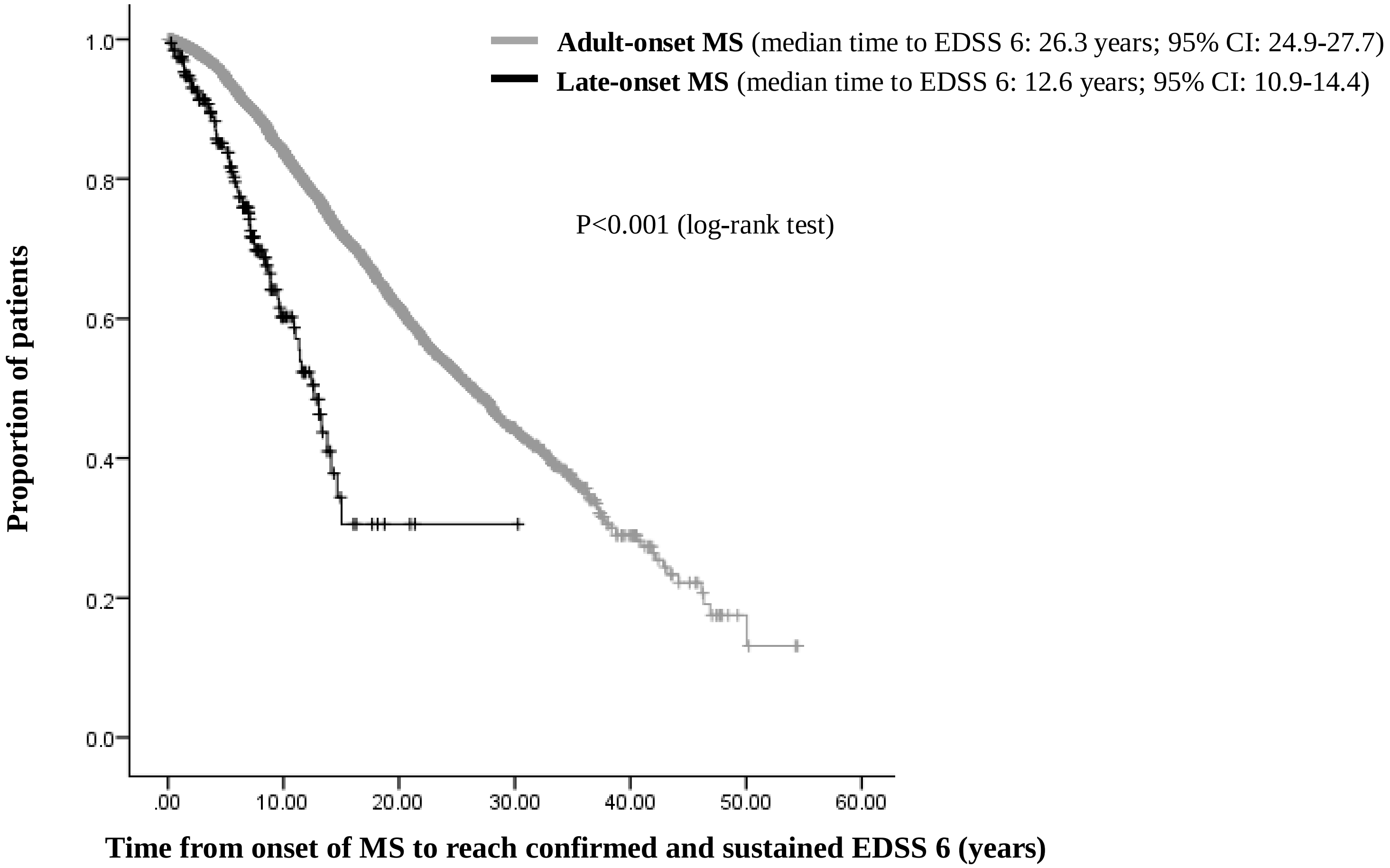

Patients at risk of progressing to EDSS 6 at selected time points

MS onset	Time from onset of MS (years)					Right-censored patients†
	0	5	10	15	20	
**Adult-onset**	4990	4108	2798	1742	1024	3525 (70.6%)
**Late-onset**	198	125	43	9	3	131 (66.2%)

Figure e-2. Panel A

Panel B: Primary-progressive MS patients (n=579) ‡

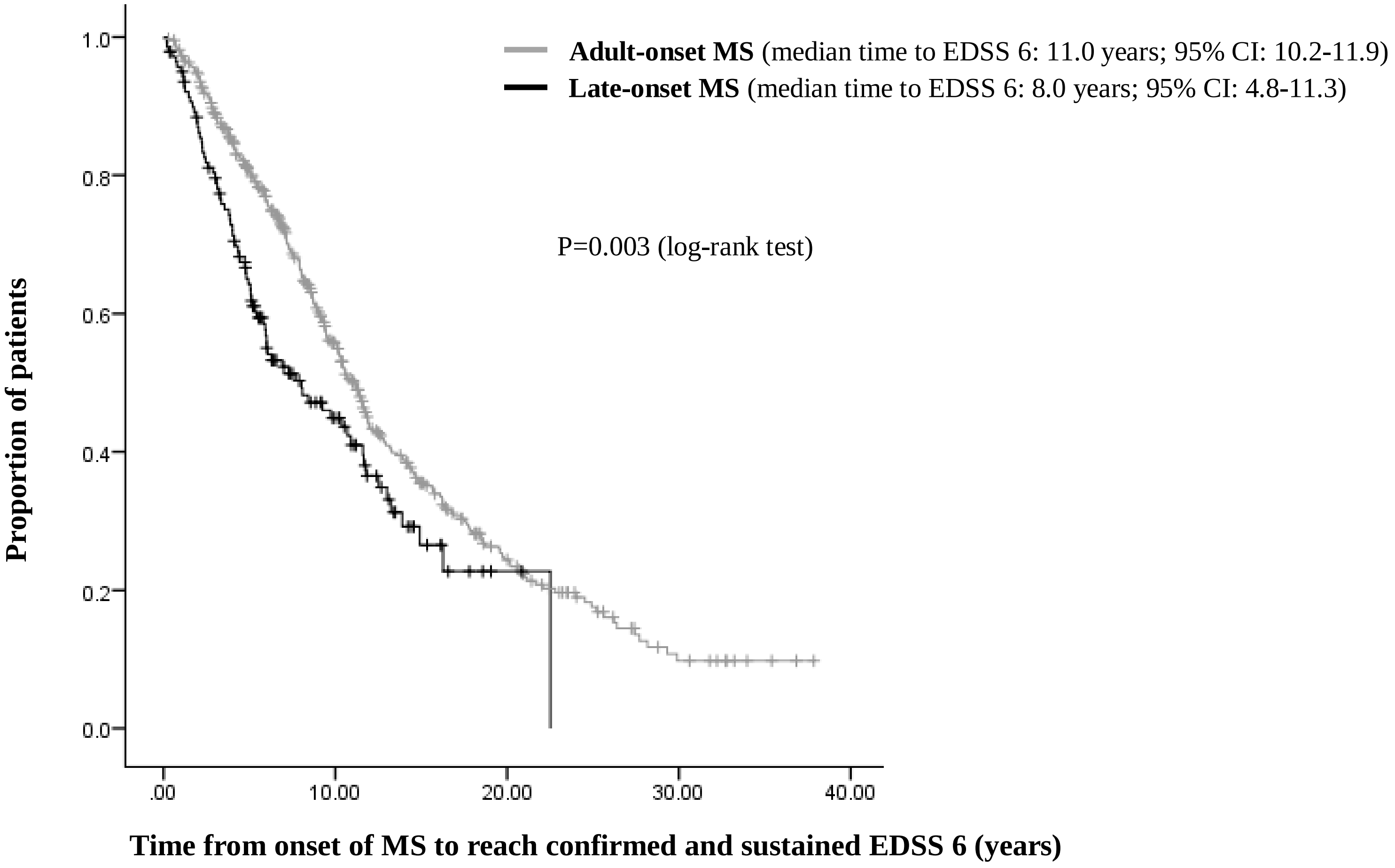

Patients at risk of progressing to EDSS 6 at selected time points

MS onset	Time from onset of MS (years)					Right-censored patients†
	0	5	10	15	20	
**Adult-onset**	438	320	184	95	51	158 (36.1%)
**Late-onset**	141	81	37	10	2	59 (41.8%)

Figure e-2. Panel B

Figure e-2. Kaplan-Meier survival curves showing time to reach confirmed and sustained EDSS 6 from onset of MS symptoms for adult-onset vs. late-onset MS patients, by disease course while including left-censored patients by assuming that these patients reached EDSS 6 at the midpoint of the interval between onset of MS symptoms and first clinic assessment (Panel A: relapsing-onset MS patients, Panel B: primary-progressive MS patients) [Aim 1].

[*] Out of 5373 patients with relapsing-onset MS, 185 patients with no EDSS score recorded did not contribute to the analysis.

[†]Those who did not reach EDSS 6 during the follow-up period.

[‡] Out of 612 patients with primary-progressive MS, 33 patients with no EDSS score recorded did not contribute to the analysis.

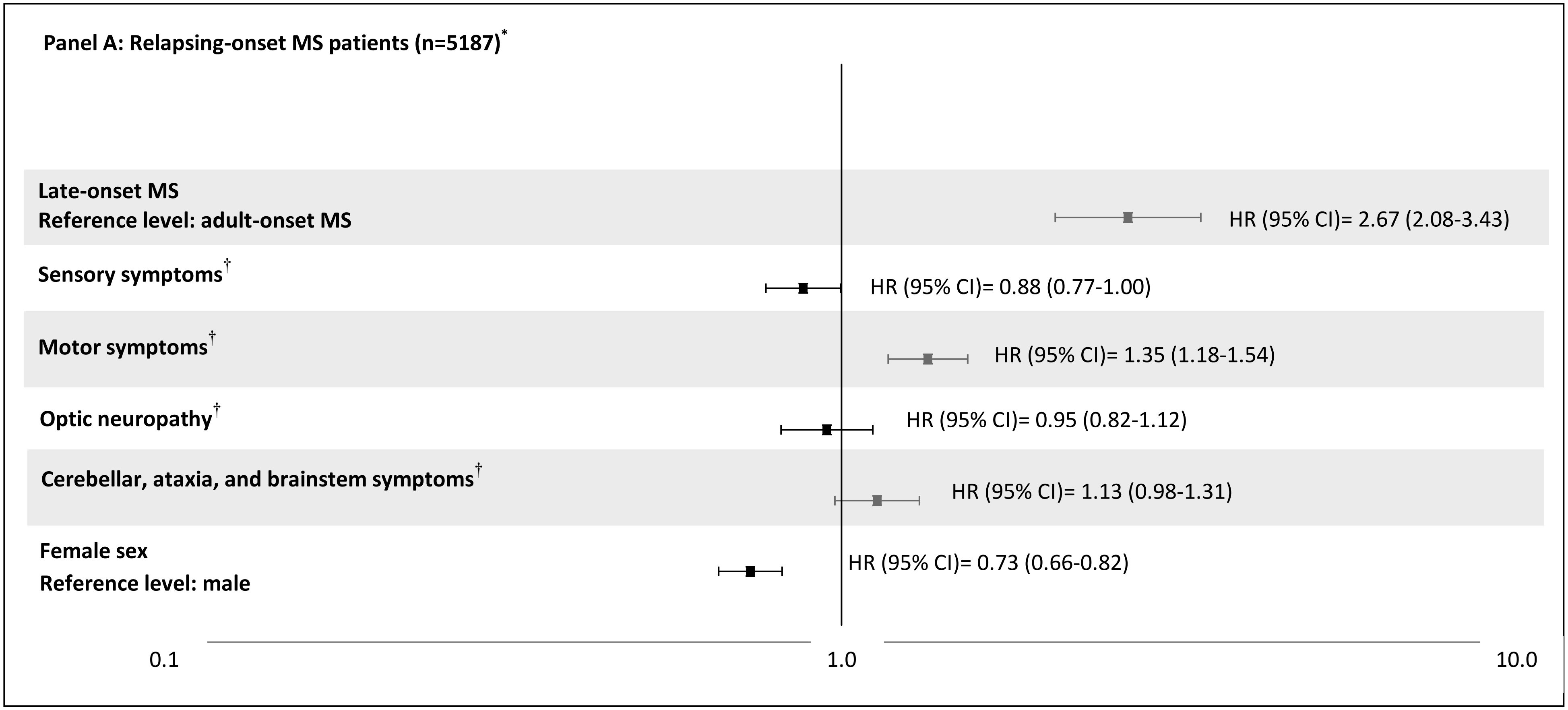

Figure e-3. Panel A

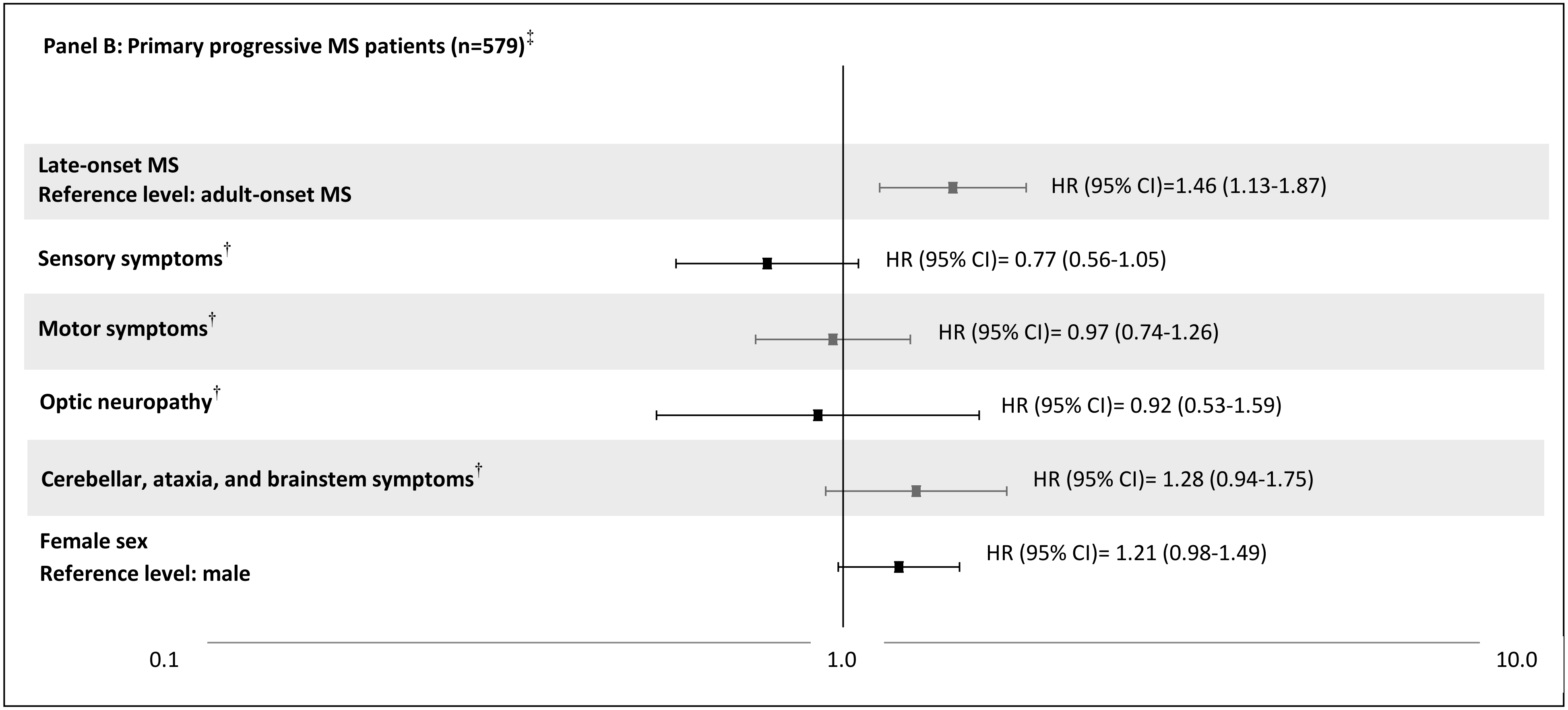

Figure e-3. Panel B

Figure e-3. Multivariable Cox regression analysis of potential factors associated with time to reach confirmed and sustained EDSS 6 from onset of MS symptoms while including left-censored patients by assuming that these patients reached EDSS 6 at the midpoint of the interval between onset of MS symptoms and first clinic assessment, for relapsing-onset MS patients (panel A) and primary-progressive MS patients (panel B) [Aim 1].

[*]Out of 5373 patients with relapsing-onset MS, 186 patients did not contribute to the analysis (including 185 patients with no EDSS score recorded, and one patient who was censored before the earliest event).

[†] Reference level= absence of the specific onset symptom.

[‡]Out of 612 patients with primary-progressive MS, 33 patients with no EDSS score recorded did not contribute to the analysis.

Relapsing-onset MS patients (n=4527)*

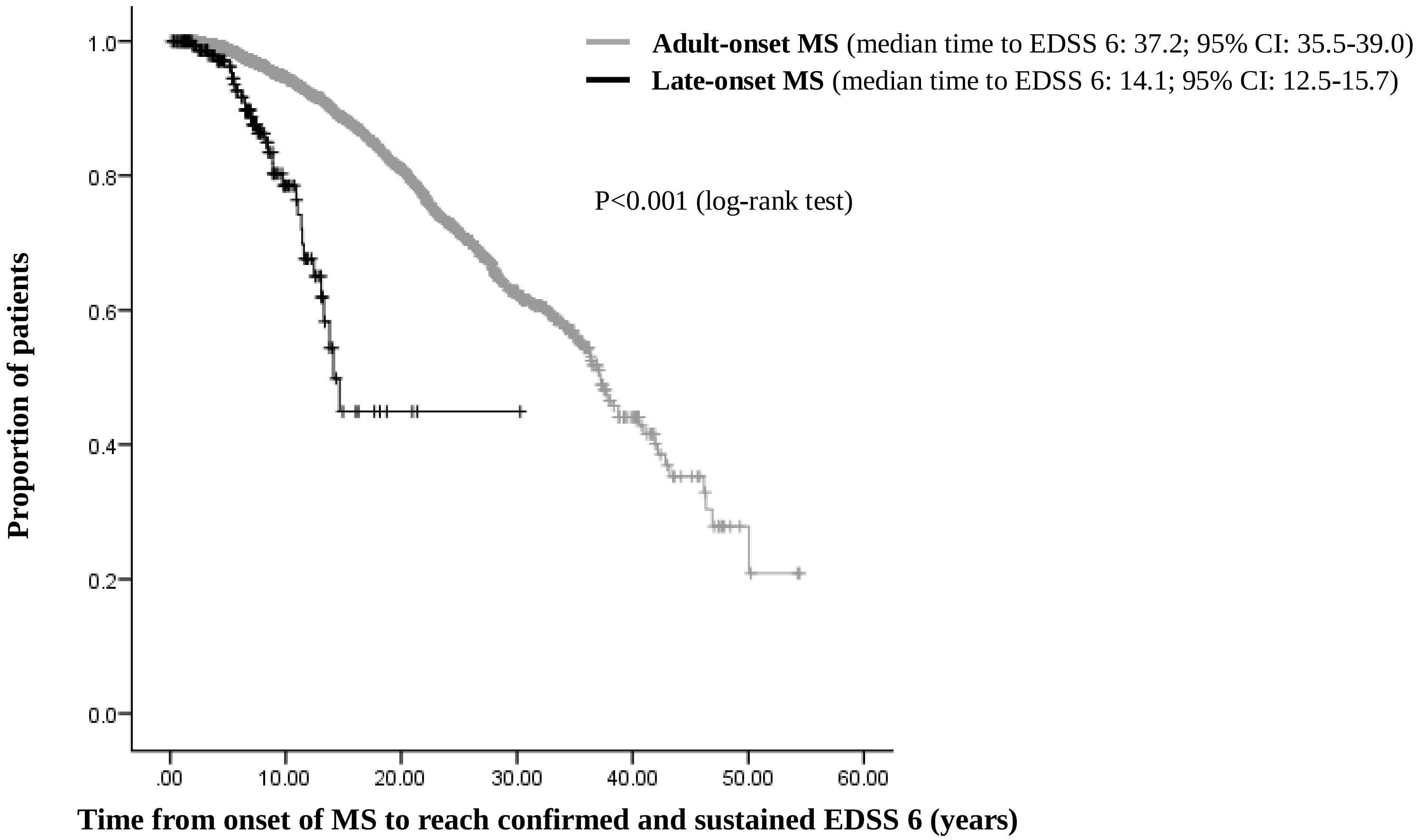

Patients at risk of progressing to EDSS 6 at selected time points

**MS onset**	**Time from onset of MS (years)**					**Right-censored patients**†
	0	5	10	15	20	
**Adult-onset**	4362	3608	2527	1620	981	3746 (85.9%)
**Late-onset**	165	112	41	8	3	134 (81.2%)

Figure e-4

Figure e-4. Kaplan-Meier survival curves of time to reach confirmed and sustained EDSS 6 from onset of MS symptoms for adult-onset vs. late-onset form of the disease among patients with relapsing-onset with data censored once a disease modifying drug was started [Aim 1].

[*] Out of 5373 patients with relapsing-onset MS, 846 patients did not contribute to the analysis (including 825 patients who had already reached the outcome by first clinic assessment did not contribute to the analysis (i.e. left-censored), and 21 patients with insufficient EDSS scores once post-treatment data was censored).

[†]Those who did not reach EDSS 6 during the follow-up period.

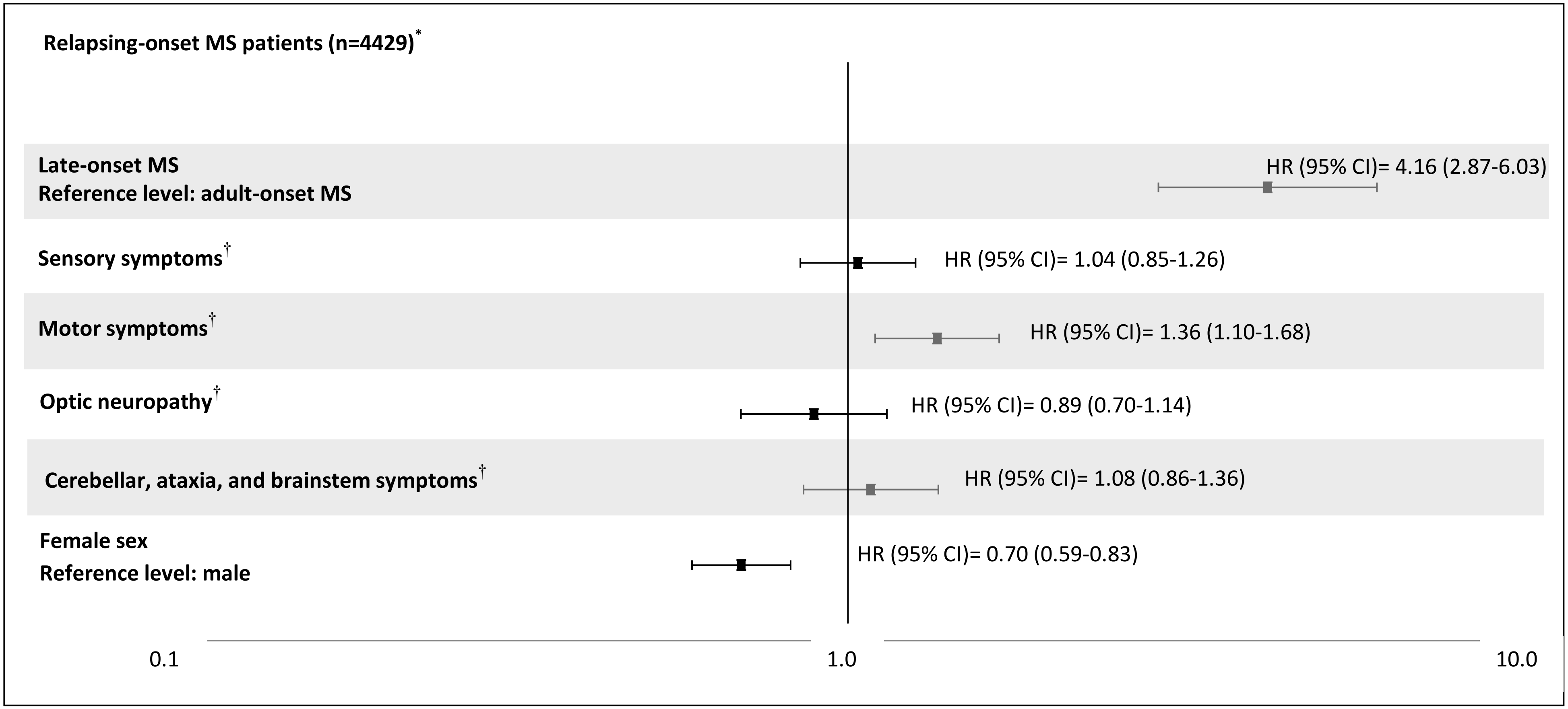

Relapsing-onset MS patients (n=4429)*
Late-onset MS
Reference level: adult-onset MS
HR (95% CI)= 4.16 (2.87-6.03)
Sensory symptoms†
HR (95% CI)= 1.04 (0.85-1.26)
Motor symptoms†
HR (95% CI)= 1.36 (1.10-1.68)
Optic neuropathy†
HR (95% CI)= 0.89 (0.70-1.14)
Cerebellar, ataxia, and brainstem symptoms†
HR (95% CI)= 1.08 (0.86-1.36)
Female sex
Reference level: male
HR (95% CI)= 0.70 (0.59-0.83)
0.1
1.0
10.0

Figure e-5

Figure e-5. Multivariable Cox regression analysis of potential factors associated with time to reach confirmed and sustained EDSS 6 from onset of MS symptoms in patients with relapsing-onset MS with data censored once a disease modifying drugs was started [Aim 1].

*Out of 5373 patients with relapsing-onset MS, 944 patients did not contribute to the analysis (including 640 patients who had already reached the outcome by first clinic assessment (i.e. left-censored), 206 patients with insufficient EDSS scores once post-treatment data was censored, and 98 patients who were censored before the earliest event).

† Reference level= absence of the specific onset symptom.

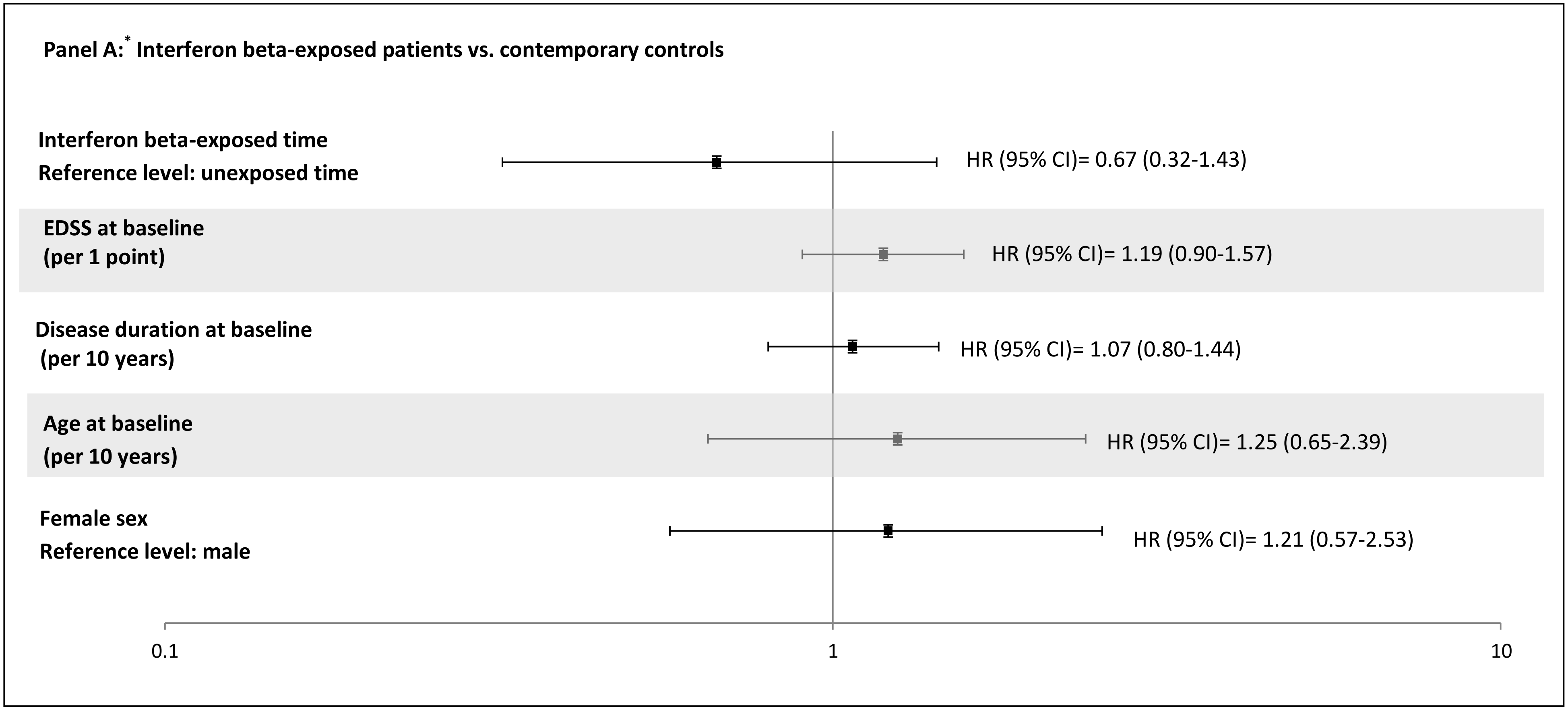

Figure e-6. Panel A

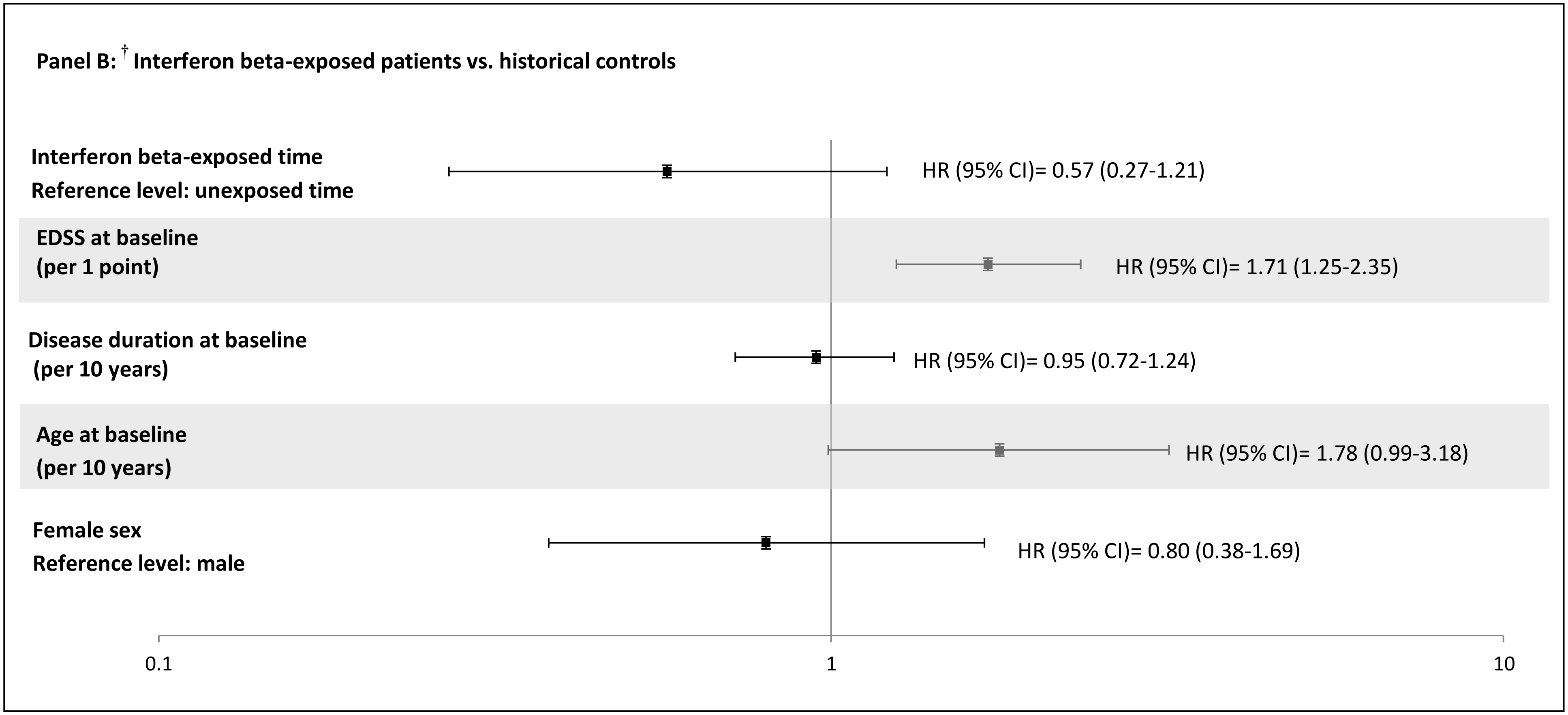

Figure e-6. Panel B

Figure e-6. Multivariable time-dependent Cox regression analysis of potential factors affecting time to reach confirmed and sustained EDSS 4 for 82 interferon beta-exposed patients vs. 155 contemporary controls aged ≥50 years at baseline, with interferon beta treatment as a time-varying covariate (panel A). Results of the same analysis for 82 interferon beta-exposed patients and 95 historical controls (panel B) [Aim 2]. The number of individuals reaching confirmed and sustained EDSS 4 were 15 (18.3%), 25 (16.1%), and 36 (37.9%) in the treated, contemporary control and historical control cohorts.

[*] Twelve patients who were censored before the earliest event did not contribute to the analysis.

[†] Ten patients who were censored before the earliest event did not contribute to the analysis.
